# Plasma cytokines IL-6, IL-8, and IL-10 are associated with the development of acute respiratory distress syndrome in patients with severe traumatic brain injury

**DOI:** 10.1186/s13054-016-1470-7

**Published:** 2016-09-15

**Authors:** Imo P. Aisiku, Jose-Miguel Yamal, Pratik Doshi, Julia S. Benoit, Shankar Gopinath, Jerry C. Goodman, Claudia S. Robertson

**Affiliations:** 1 Department of Emergency Medicine, Brigham and Women’s Hospital, 75 Francis Street, Boston, MA 02115 USA; 2 Division of Biostatistics, University of Texas School of Public Health, Houston, TX USA; 3 Department of Emergency Medicine and Internal Medicine, University of Texas Health Science Center at Houston, Houston, TX USA; 4 Department of Basic Vision Sciences, College of Optometry Texas Institute for Measurement Evaluation and Statistics, University of Houston, Houston, TX USA; 5 Department of Neurosurgery, Baylor College of Medicine, Houston, TX USA; 6 Department of Pathology, Baylor College of Medicine, Houston, TX USA

**Keywords:** TBI, ARDS, ALI, Cytokines, Plasma, CSF

## Abstract

**Background:**

Patients with severe traumatic brain injury (TBI) are at risk of the development of acute respiratory distress syndrome (ARDS). TBI and ARDS pathophysiologic mechanisms are known to independently involve significant inflammatory responses. The literature on the association between plasma inflammatory cytokines and ARDS in patients with TBI is sparse.

**Methods:**

The study was a secondary analysis of the safety of a randomized trial of erythropoietin and transfusion threshold in patients with severe TBI. Inflammatory markers within the first 24 hours after injury were compared in patients who developed ARDS and patients without ARDS, using Cox proportional hazards models.

**Results:**

There were 200 patients enrolled in the study. The majority of plasma and cerebrospinal fluid (CSF) cytokine levels were obtained within 6 hours. Plasma proinflammatory markers IL-6 and IL-8 and anti-inflammatory marker IL-10 were associated with the development of ARDS (adjusted hazard ratio (HR) = 1.55, confidence interval (CI) = 1.14, 2.11, *P* = 0.005 for IL-6; adjusted HR = 1.32, CI = 1.10, 1.59, *P* = 0.003 for IL-8).

**Conclusion:**

Plasma markers of IL-6, IL-8, and IL-10 are associated with ARDS in patients with severe TBI.

**Trial registration:**

NCT00313716 registered 4/2006

## Background

Severe head injury is associated with an early inflammatory response of which proinflammatory and anti-inflammatory cytokines are critical mediators of neuro-inflammation [1–4]. The extent of brain injury is determined by the severity of the primary mechanical injury and the consequences of the secondary biomolecular injury patterns [1–8]. Proinflammatory markers but not anti-inflammatory markers have been found to be upregulated in serum from patients with TBI [9]. Some inflammatory markers such as IL-6 have been reported to be a possible predictor of elevated intracranial pressure (ICP) in patients with isolated TBI [10]. These reports support an inflammatory component to the pathophysiology of severe TBI.

Acute respiratory distress syndrome (ARDS) is known to involve an intense proinflammatory and anti-inflammatory response in the lungs as demonstrated in numerous studies [11–14]. Decreased IL-10 in bronchoalveolar lavage fluid has been associated with mortality in patients with ARDS [15]. Although there is a clear relationship between inflammatory mediators and ARDS, elevated plasma cytokines have not been shown to be associated with ARDS in at-risk medical patients [16, 17]. None of these studies included patients with TBI.

Patients with severe TBI have high mortality and ARDS is recognized as one of the significant contributors to in-patient mortality and prolonged mechanical ventilation. Secondary injury may involve the development of systemic inflammatory response syndrome (SIRS), ARDS, multiple organ dysfunction syndrome, or sepsis [18–20]. Recognizing that TBI and ARDS involve significant inflammatory mediators and patients with severe TBI are at risk of ARDS, we hypothesized that early proinflammatory and anti-inflammatory cytokines may be associated with the development of ARDS.

## Methods

### Primary hypothesis and outcomes

The objective of this study was to determine the association between cytokine levels just after severe traumatic brain injury and the risk of ARDS. The Berlin definition of ARDS events was used [21], based on acute onset within 7 days, bilateral pulmonary infiltrates consistent with pulmonary edema, impaired oxygenation (partial pressure of oxygen (PaO2)/inspired oxygen fraction (FiO2) ratio <300 mm Hg), and impaired oxygenation not fully explained by cardiac failure [21].

### Study design

This is a secondary analysis of the ARDS safety outcome from the erythropoietin randomized controlled trial. The study design has been reported previously [22]. Briefly, participants were randomly assigned to administration of erythropoietin or placebo and to hemoglobin transfusion thresholds of 7 or 10 g/dl in a 2 × 2 factorial design. Participants with a closed head injury who were not able to follow commands after resuscitation and could be enrolled within 6 hours of injury were recruited from two level-1 trauma centers. Patients were excluded if their Glasgow Coma Scale (GCS) score was 3, and they had fixed and dilated pupils, penetrating trauma, pregnancy, life-threatening systemic injuries, or severe preexisting disease. Transfusion of leuko-reduced packed red blood cells was used to maintain the assigned hemoglobin threshold. Patients were enrolled using the emergency consent exception when relatives were not available for prospective consent. The study was approved by the local Institutional Review Boards (IRBs) at participating institutions.

### Cytokine and nitric oxide (NOx) measurements

Heparinized blood samples were collected, usually via a radial artery catheter. The samples were centrifuged, aliquoted, and temporarily stored at –80 °C in an area adjacent to the ICUs at the hospitals until they were transferred to our laboratory at Baylor College of Medicine for analysis. Both enrolling hospitals were adjacent to Baylor and the samples were transferred on dry ice from the freezer at the hospital to the freezer at Baylor where they were kept until they were analyzed.

Cytokine measurements were performed using multiplex flow cytometric bead array analysis (Beckton Dickinson Cytometric Bead Array, Human Inflammation Kit, San Jose, CA, USA) in the Cytometry and Cell Sorting Core at Baylor College of Medicine. This method permits the simultaneous measurement of six cytokines (IL-1β, IL-6, IL-8, IL-12, TNFα, and IL-10) in a single sample. In this study, we evaluated IL-1β, IL-6, IL-8, IL-12, TNFα, IL-10 and nitric oxide (NOx). The final products of NO oxidation (nitrate (NO_3_-) and nitrite (NO_2_-)) were analyzed in plasma samples that were ultra-filtered through a 30-kDa molecular weight cutoff filter (Millipore Corporation, Billeria, MA, USA), and then transferred to a plate to be analyzed using a Nitrate/Nitrite Fluorometric assay kit (Cayman Chemical’s Company, Ann Harbor, MI, USA) using an excitation wavelength of 360–365 nm and an emission wavelength of 430 nm (Synergy 2 Multi-Mode Microplate Reader Biotek Instruments, Inc).

### Data analysis

Statistical analyses were performed using SAS version 9.3 (SAS Institute Inc) and R version 2.13.1 (R Foundation for Statistical Computing) with two-sided statistical tests at a 0.05significance level. Baseline covariates considered were transfusion threshold randomization group (<10 g/dl; <7 g/dl); erythropoietin (EPO) randomization group (Epo1 or Epo2 if randomized to the first or second erythropoietin dosing regimens, respectively, versus placebo); pre-hospital hypotension present; pre-hospital hypoxia present; age (years); emergency room (ER) GCS-sum; Injury Severity Score (ISS); number of unreactive pupils (one or two versus none); ER computed tomography (CT) high risk; and intubated in ER (versus ICU or in the field) at baseline. The first measurement of cytokines within 24 hours after injury was used as the baseline value. Although some measurements were not obtained before transfusion or before EPO administration, there was no difference in ALI/ARDS between the intervention and control arms of the randomized trial [22].

The Wilcoxon rank sum test or Fisher’s exact test was used to compare continuous and categorical variables, respectively, among patients who did or did not develop ARDS. Cox proportional hazards regression was used to determine whether the cytokine values were associated with an increased risk of ARDS, adjusted for baseline variables. Due to the large number of potential baseline variables and the relatively small number of ARDS events, variables were selected to build a parsimonious model. Lasso-penalized Cox [23] regression, with the penalty parameter selected using fivefold cross-validation, was used for selection of variables in addition to the transfusion threshold randomization group and all baseline variables that were significantly different among patients who did or did not have an ARDS event. The lasso penalization has better statistical properties than other common methods for variable selection, such as forward or backward stepwise selection, which tend to result in underestimation of the standard errors of the coefficients. Censor time was defined as 6 months after injury, date of hospital discharge, death, or withdrawal, whichever occurred first. Spearman’s rank correlation (rho) was used to assess the correlation between the initial cytokine value and the category of ARDS severity based on the Berlin definition of ARDS.

## Results

**Fig. 1:**
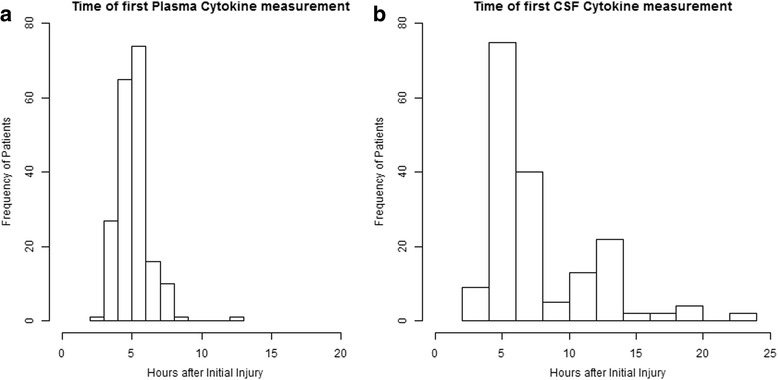
**a** Distribution of time of plasma cytokine measurement after injury. **b** Distribution of time of cerebrospinal fluid cytokine measurement after injury

A total of 200 patients were enrolled in the study of whom between 173 and 195 (87–98 %) were included in the cytokine analyses, as there were varying numbers of missing values for each cytokine. Patients were excluded if they did not have any cytokine values available within 24 hours of injury or if they had an ARDS event on the first day, because we did not know whether the cytokine measurement was obtained before or after the ARDS event. The baseline clinical characteristics are presented in Table 1. There were some significant differences in some baseline characteristics between patients with and without ARDS, notably a significant difference in gender (96.2 % male (50/52) vs. 83.1 % (123/148), respectively, *p* = 0.03). The GCS sum score in the ER (median = 6 vs. 7, *p* = 0.02), Acute Physiology and Chronic Health Evaluation (APACHE) II score (24 vs. 19, *p* <0.001), Abbreviated Injury Severity Score (median = 32 vs. 28, *p* = 0.01), and pre-hospital hypoxia (present in 30.8 % of patients (16/52) vs. 15.5 % (23/148)), respectively. The majority (86 %) of plasma cytokines were collected within 6 hours of injury (Fig. 1a) and 48 % of CSF cytokines were collected within 6 hours of injury (Fig. 1b).

**Table 1:** Baseline clinical characteristics by incident

Characteristic	Developed acute respiratory distress syndrome (Berlin definition)	Did not develop acute respiratory distress syndrome (Berlin definition)	*P* value
	(*n* = 52)	(*n* = 148)	
Age (years), median (IQR)	29.0 (19.5)	30.0 (23.0)	0.87
Male sex, *n* (%)	50 (96.2)	123 (83.1)	0.03
Race, *n* (%)			
black	8 (15.4)	35 (23.6)	0.44
white	16 (30.8)	32 (21.6)	
Hispanic	27 (51.9)	76 (51.4)	
Asian	1 (1.9)	5 (3.4)	
Emergency room sum Glasgow Coma Scale			
> 8	7 (13.5)	38 (25.7)	0.04
6–8	21 (40.4)	68 (45.9)	
3–5	24 (46.2)	42 (28.4)	
Emergency room sum Glasgow Coma Scale, median (IQR)	6.0 (5.0)	7.0 (4.0)	0.02
Marshall classification on computed tomography			
mild diffuse injury I–II	23 (44.2)	66 (44.6)	0.22
severe diffuse injury III–IV	16 (30.8)	30 (20.3)	
mass lesion	13 (25.0)	52 (35.1)	
Acute Physiology and Chronic Health Evaluation II score, median (IQR)	24.0 (11.0)	19.0 (7.0)	<0.001
Abbreviated Injury Severity Score, median (IQR)	32.0 (14.5)	28.0 (10.0)	0.01
Intracranial pressure (*n* = 197), median (IQR)	14.0 (12.0)	14.0 (12.0)	0.84
Mechanism of injury, *n* (%)			
assault	7 (13.5)	15 (10.1)	0.93
fall/jump	7 (13.5)	20 (13.5)	
motor vehicle	28 (53.8)	88 (59.5)	
Motorcycle	9 (17.3)	22 (14.9)	
Other	1 (1.9)	3 (2.0)	
Surgery on admission, *n* (%)	14 (26.9)	57 (38.5)	0.18
Pre-hospital hypotension, *n* (%)	7 (13.5)	18 (12.2)	0.81
Pre-hospital hypoxia	16 (30.8)	23 (15.5)	0.02
Time (days) from injury to ALI, median (IQR)	3.0 (3.0)

Plasma and CSF cytokine levels by ARDS status are presented in Tables 2 and 3. Median IL-6, IL-8, and IL-10 (IL-6 = 568.2, IL-8 = 158.1, and IL-10 = 14.5 pg/mL) in plasma from the ARDS group was significantly higher than in the non-ARDS group (IL-6 = 241.2, IL_8 = 57.4, and IL-10 = 10.0 pg/mL). In CSF, median IL-10 was 7.0 and 5.1 pg/mL in patients with and without ARDS, respectively, with a *p* value of 0.026.

**Table 2:** Comparison of plasma cytokines among patients who had or did not have ARDS

Cytokine	Normal ranges (pg/mL)	ARDS median (25^th^, 75^th^) (min–max)	No ARDS median (25^th^, 75^th^) (min–max)	*P* value
		(*n* = 51)	(*n* = 144)	
IL-12p70 (n = 194)	0–6	3.9 (2.2, 5.9) (0.1–163.6)	3.8 (2.7, 5.4) (0.2–40.7)	0.882
IL-10 (n = 195)	0–18	14.5 (8.1, 34.8) (0.2–147.6)	10.0 (6.1, 18.6) (0.2–98.2)	0.008
IL-8 (n = 194)	0–5	158.1 (45.0, 284.0) (0.04–2504.0)	57.4 (26.0, 135.1) (0.1–7393.0)	<0.001
IL-6 (n = 195)	0–5	568.2 (224.4, 2278.0) (5.9–18,950)	241.2 (92.7, 638.8) (3.0–21,330.0)	<0.001
IL-1β (n = 195)	0–39	5.4 (3.1, 9.0) (0.1–54)	4.7 (3.2, 8.3) (0.1–142.8)	0.702
Nitric oxide (n = 193)	Not available	1.7 (1.3, 2.5) (0.01– 14.8)	1.5 (1.0, 2.4) (0.01–7.3)	0.266
TNF-α (n = 195)	0–22	3.8 (1.8, 6.2) (0.1–38.8)	3.9 (3.2, 5.4) (0.3– 95.9)	0.743

**Table 3:** Comparison of cerebrospinal fluid cytokines among patients who did or did not have acute respiratory distress syndrome (ARDS) and those who did not

Cytokine	Normal ranges (pg/mL)	ARDS median (25^th^, 75^th^) (min–max)	No ARDS median (25^th^, 75^th^) (min–max)	*P* value
		(*n* = 45)	(*n* = 129)	
IL-12p70 (*n* = 173)	0–2	3.8 (2.3, 5.7) (0.1–54.4)	3.5 (0.9, 4.1) (0.1–45.9)	0.155
IL-10 (*n* = 174)	0–1.5	7.0 (4.3, 15.6) (0.2–78.4)	5.1 (3.5, 10.0) (0.2–303.7)	0.026
IL-8 (*n* = 173)	0–2	1203.0 (93.7, 4782.0) (3.9–32,570.0)	985.9 (72.6, 6792.0) (0.04–204,900)	0.716
IL-6 (*n* = 174)	0–1	1820.0 (233.3, 7083.0) (0.2–17,790.0)	609.4 (58.6, 2917.0) (0.2–76,370)	0.085
IL-1β (*n* = 174)	0–3	5.6 (3.7, 8.0) (0.1–74.6)	5.2 (3.5, 8.1) (0.1–82.4)	0.490
Nitric oxide (*n* = 172)	Not available	0.5 (0.3, 0.9) (0.01–3.6)	0.5 (0.1, 0.7) (0.01–3.98)	0.198
TNF-α (*n* = 174)	0–2	4.2 (2.1, 6.3) (0.3–38.8)	3.8 (2.0, 4.3) (0.3–30.3)	0.179

Cox proportional hazard ratios (HR) for time to ARDS are shown in Tables 4 and 5. We observed 66 % higher risk for each unit increase in plasma IL-10 (HR = 1.66, 95 % CI = 1.22, 2.26, *p* = 0.001). Plasma IL-6 and IL-8 were associated with 24 % and 26 % higher risk, respectively, for each unit increase in cytokine value (IL-6 adjusted HR = 1.24, 95 % CI = 1.05. 1.49, *p* = 0.012; IL-8 adjusted HR = 1.26, 95 % CI = 1.04, 1.49, *p* = 0.02). CSF IL-12 was associated with 52 % increased risk for each unit increase (adjusted HR = 1.52, 95 % CI = 1.05, 2.21, *p* = 0.03). CSF IL-10 was associated with 33 % increased risk for each unit increase and was statistically significant (adjusted HR = 1.33, 95 % CI = 1.00, 1.76, *p* = 0.05). There were no significant differences in other proinflammatory and anti-inflammatory markers in either the plasma or CSF (Tables 2, 3, 4 and 5). There was positive correlation between the initial plasma cytokine value and the Berlin definition of ARDS by severity: for IL-6 rho = 0.12, *p* < 0.001, for IL-8 rho = 0.14, *p* < 0.001, and for IL-10 rho = 0.15, *p* < 0.001.

**Table 4:** Associations between each plasma cytokine and time to development of acute respiratory distress syndrome from Cox regression analysis in erythropoietin study subjects with traumatic brain injury (unadjusted and adjusted analyses)

		Unadjusted			Adjusted model		
	Number	HR	95 % CI	*P* value	HR	95 % CI	*P* value
IL-12p70	194	1.03	0.74,0.14	0.88	1.18	0.82, 1.69	0.36
TT10					1.24	0.70, 2.19	0.46
ER sum GCS					0.86	0.76, 0.98	0.02
Pre-hospital hypoxia					1.71	0.88, 3.33	0.11
Male sex					4.97	1.17, 21.16	0.03
ISS					1.04	1.00, 1.07	0.03
Intubated in ER					1.51	0.72, 3.17	0.28
Pre-hospital hypotension					0.62	025, 1.56	0.31
Pupil unreactive (1–2 vs. 0)					0.65	0.35, 1.22	0.18
TNF-α	195	0.96	0.67,1.37	0.81	1.03	0.71, 1.51	0.85
TT10					1.17	0.067, 2.07	0.56
ISS					1.03	0.99, 1.07	0.05
Male sex					4.79	1.15, 20.06	0.03
ER sum GCS					0.86	0.76, 0.98	0.02
Pre-hospital hypoxia					1.78	0.92, 3.43	0.08
Pre-hospital hypotension					0.62	025, 1.54	0.30
Pupil unreactive (1–2 vs. 0)					0.67	0.36, 1.25	0.21
Intubated in ER					1.55	0.74, 3.25	0.25
IL-10	195	1.57	1.17,2.11	<0.01	1.66	1.22, 2.26	<0.01
TT10					1.21	0.69, 2.11	0.51
ER sum GCS					0.86	0.76, 0.98	0.02
Pre-hospital hypoxia					2.00	0.98, 3.73	0.06
Male sex					5.23	1.23, 22.29	0.03
ISS					1.03	0.99, 1.06	0.11
Pre-hospital hypotension					0.50	0.20, 1.23	0.13
Pupil unreactive (1–2 vs. 0)					0.56	0.30, 1.05	0.07
IL-6	195	1.30	1.11,1.52	<0.01	1.24	1.05, 1.49	0.01
TT10					1.14	0.65, 1.99	0.65
Pre-hospital hypoxia					1.64	0.85, 3.16	0.14
Male sex					4.75	1.13, 19.95	0.03
ER sum GCS					0.89	0.79, 1.01	0.06
ISS					1.02	0.99, 1.06	0.22
Intubated in ER					1.50	0.71, 3.18	0.29
Pre-hospital hypotension					0.52	0.21, 1.28	0.15
Pupil unreactive (1–2 vs. 0)					0.60	0.32, 1.12	0.11
IL-1β	195	0.98	0.73,1.30	0.87	0.98	0.73, 1.32	0.89
TT10					1.17	0.67, 2.05	0.58
ISS					1.03	1.00, 1.07	0.06
ER sum GCS					0.86	0.76, 0.98	0.02
Pre-hospital hypoxia					0.62	0.25, 1.55	0.09
Pupil unreactive (1–2 vs. 0)					0.67	0.36, 1.25	0.21
Male sex					4.75	1.13, 20.0	0.03
Intubated in ER					1.57	0.74, 3.30	0.24
Pre-hospital hypotension					0.62	0.25, 1.55	0.31
IL-8	194	1.33	1.11,1.59	<0.01	1.26	1.04, 1.53	0.02
TT10					1.04	0.58, 1.87	0.89
ISS					1.02	0.98, 10.5	0.62
ER sum GCS					0.90	0.80, 10.2	0.10
Pre-hospital hypoxia					1.94	1.01, 3.74	0.05
Pupil unreactive (1–2 vs. 0)					0.61	0.33, 1.15	0.12
Male Sex					4.20	1.01, 17.53	0.05
Pre-hospital hypotension					0.55	0.22, 1.35	0.19
NOx	193	1.64	0.90,3.00	0.11	1.60	0.89, 2.90	0.12
TT10					1.12	0.64, 1.97	0.69
ISS					1.03	1.00, 1.06	0.06
ER sum GCS					0.91	0.81, 1.03	0.13
Pre-hospital hypotension					0.56	0.22, 1.41	0.22
Pre-hospital hypotension					1.84	0.95, 3.56	0.07
Male sex					4.73	1.14, 19.62	0.03

Variables considered for adjustment included age, injury severity score (ISS), emergency room (ER) sum Glasgow Coma Scale (GCS), unreactive pupils, emergency room computer tomography indicating high risk, pre-hospital hypotension, pre-hospital hypoxia, intubated in the ER, transfusion threshold, and each baseline cytokine level. Cytokine level, transfusion threshold, pre-hospital hypoxia, ER sum GCS, sex, and ISS were forced into the adjusted model. *HR* hazard ratio, *IL-12p70* interleukin-12 p70, *TNF-α* tumor necrosis factor alpha, *IL-10* interleukin-10, *IL-6* interleukin-6, *IL-1β* interleukin-1 beta, *IL-8* interleukin-8, *NOx* nitric oxide, *TT10* transfusion threshold <10 g/dL

**Table 5:** Associations between each cytokine measured in cerebrospinal fluid and time to development of acute respiratory distress syndrome from Cox regression analysis in erythropoietin study subjects with traumatic brain injury (unadjusted and adjusted analyses)

		Unadjusted			Adjusted model		
	Number	HR	95 % CI	*P* value	HR	95 % CI	*P* value
IL-12p70	173	1.36	0.94, 1.95	0.10	1.52	1.04, 2.21	0.03
TT10					1.12	0.62, 2.04	0.69
Pre-hospital hypoxia					1.38	0.68, 2.79	0.38
Male sex					8.69	1.17, 64.53	0.03
ER sum GCS					0.90	0.79, 1.03	0.11
ISS					1.04	1.00, 1.07	0.04
TNF-α	174	1.22	0.83, 1.80	0.32	1.43	0.97, 2.14	0.08
TT10					1.08	0.60, 1.95	0.80
Pre-hospital hypoxia					1.27	0.63, 2.58	0.51
Male sex					10.40	1.38, 78.61	0.02
ER sum GCS					0.86	0.75, 0.99	0.03
ISS					1.04	1.01, 1.08	0.02
IL-10	174	1.27	0.99, 1.64	0.06	1.33	1.00, 1.76	0.05
TT10					1.27	0.69, 2.35	0.43
Pre-hospital hypoxia					1.57	0.64, 0.79	0.20
Male sex					6.98	0.95, 51.31	0.06
ER sum GCS					0.87	0.76, 0.99	0.04
ISS					1.02	0.99, 1.05	0.18
IL-.6	174	1.09	0.97, 1.21	0.14	1.06	0.95, 1.19	0.28
TT10					1.06	0.59, 1.92	0.83
Pre-hospital hypoxia					1.41	0.70, 2.82	0.33
Male sex					7.60	1.04, 55.59	0.05*
ER sum GCS					0.91	0.81, 1.03	0.15
ISS					1.02	0.99, 1.06	0.14
IL-1β	174	1.05	0.77, 1.43	0.76	1.11	0.80, 1.54	0.54
TT10					0.91	0.60, 2.01	0.76
Pre-hospital hypoxia					1.30	0.64, 2.65	0.47
Male sex					9.25	1.24, 69.18	0.03
ER sum GCS					0.88	0.78, 1.01	0.06
ISS					1.04	1.00, 1.08	0.14
IL-8	173	1.02	0.92, 1.13	0.70	1.01	0.92, 1.12	0.81
TT10					1.11	0.61, 2.01	0.73
Pre-hospital hypoxia					1.42	0.70, 2.88	0.32
Male sex					7.68	1.05, 56.22	0.04
ER sum GCS					0.92	0.81, 1.04	0.19
ISS					1.03	0.99, 1.06	0.11
NOx	172	2.11	0.89, 0.502	.09	1.66	0.70, 3.97	0.25
TT10					1.17	0.64, 2.13	0.61
Pre-hospital hypoxia					1.39	0.67, 2.88	0.37
Male sex					6.76	0.91, 49.9	0.06
ER sum GCS					0.89	0.78, 1.02	0.09
ISS					1.02	0.99, 1.06	0.14

Variables considered for adjustment included age, injury severity score (ISS), enrollment sum Glasgow Coma Scale (GCS), unreactive pupils, emergency room (ER) computed tomography indicating high risk, pre-hospital hypotension, pre-hospital hypoxia, intubated in the ER, transfusion threshold, and each baseline cytokine level. Cytokine level, transfusion threshold, pre-hospital hypoxia, ER sum GCS, sex, and ISS were forced into the adjusted model. *HR* hazard ratio, *IL-12p70* interleukin-12 p70, *TNF-α* tumor necrosis factor alpha, *IL-10* interleukin-10, *IL-6* interleukin-6, *IL-1β* interleukin-1 beta, *IL-8* interleukin-8, *NOx* nitric oxide, *TT10* transfusion threshold <10 g/dL

High tidal volume ventilator strategies are known to induce volume trauma and barotrauma and low tidal volume strategies may be protective in patients with ARDS. The median initial tidal volume, i.e., prior to the development of ARDS, was 8.52 mL/kg (25^th^–75^th^ percentiles = 8.18–9.10) for patients who developed ARDS (*n* = 35) and 9.02 mL/kg (25^th^–75^th^ percentiles = 8.38–9.76) for patients who did not develop ARDS (*n* = 93), and this was statistically significantly different (*p* = 0.04).

## Discussion

Acute respiratory distress syndrome is a well-described complication in patients who survive an initial traumatic insult. Inflammatory responses to TBI, both local and systemic, are complex. Invading cells from the blood (e.g., leukocytes, monocytes and macrophages) and/or activated resident cells (e.g., microglia) release a number of inflammatory molecules and free radicals that can contribute to brain edema and worsen neurological outcome. Increased serum and/or CSF concentrations of proinflammatory and anti-inflammatory cytokines, chemokines, and acute phase reactant proteins have been observed following TBI. The importance of inflammation in the progression of TBI-associated pathologic change has been the focus of numerous studies [1, 24, 25]. More recently, the expression of these factors has been appreciated as a potential marker of injury, and studies have been carried out to test whether these factors correlate with outcome or can serve as surrogate markers of treatment efficacy.

TBI biomarkers have primarily focused on prediction of elevated intracranial pressure or mortality. Several biomarkers have emerged as possible markers of significant neurological injury, including s100B, IL-8, and ceruloplasmin. There are no data on biomarkers that predict ARDS in patients with severe TBI, despite incidence of 20–30 % and high mortality. In two separate studies in the late 1990s [16, 17], Parsons and Pittet did not identify any association with inflammatory cytokines in non-TBI in at-risk patients with ARDS.

Our study is the first to identify correlation between initial cytokines in patients with TBI and the development of ARDS. Elevated plasma markers of proinflammatory cytokines IL-6 and IL-8 and anti-inflammatory marker IL-10 may indicate the importance of an extracranial inflammatory response in the development of ARDS. CSF cytokines IL-12 and IL-10 were statistically significant in a fully adjusted model for markers of severity of illness, which we report in Table 5. CSF IL-12 and IL-10 are potentially correlated with ARDS in patients with TBI, but is difficult to confidently interpret this result and we suggest the results be cautiously interpreted and that additional studies may be required to confirm these associations. Severe TBI likely induces an inflammatory response that is not limited to the brain and the TBI-mediated systemic response is likely involved in the development of ARDS, and may be quantifiable in the plasma. This is particularly relevant clinically and in research, as early plasma markers are easier to obtain than CSF.

The majority of cytokine levels were drawn within 6 hours of injury. The time frame within which the cytokines were measured suggests an inflammatory response that is triggered from the initial intracranial injury. Interestingly, elevated ICP, a known prognosticator of mortality and a marker of disease severity in patients with severe TBI [26], and the Marshall CT classification of injury were similar in the ARDS and non ARDS groups and therefore did not impact the development of ARDS. It is worth noting that other potential triggers of an inflammatory response were considered, including mechanical ventilation and transfusion. High tidal volume mechanical ventilation is known to be deleterious and contribute to ventilator-induced lung injury (VILI) [27, 28]. Patients with severe TBI frequently have elevated ICP, which makes low tidal volume lung-protective ventilation [29] and permissive hypercapnea challenging strategies to employ. The tidal volume in our study population was modest and below typical thresholds for high tidal-volume ventilation. Ventilator strategy likely did not contribute to the incidence of ARDS as there was no difference between the two groups. Critical to the design of the study was the randomization of patients to prespecified transfusion triggers. The baseline cytokine values reflect the period before administration of any blood products, and we have previously reported [22] that there was no difference in the incidence of ARDS between the intervention and control groups. Physicians caring for patients with severe TBI should be aware of the complex neuroinflammatory mechanisms that are associated with ARDS in patients with TBI and should consider strategies to minimize lung injury early in the treatment algorithm in patients who survive the initial injury.

Our study has several limitations worth acknowledging. The study is a retrospective and secondary analysis of prospectively collected data on erythropoietin in severe TBI. However, we feel this does not impact the results or strength of the study as the variables and outcomes were selected and collected prospectively. The cytokine measurements were single as opposed to sequential measurements. While sequential measurements may provide additional information, we felt sequential measurements would be more difficult to interpret and could potentially be influenced by our primary interventional study. Therefore, initial cytokine concentrations were used for analysis. Our study protocol did not require lung-protective strategies in all patients. However, there was no statistical difference in mean tidal volume between our groups and we do not believe this contributed to the development of ARDS.

## Conclusion

Elevated plasma inflammatory markers IL-6, IL-8, and IL-10 in the early phase of severe acute TBI are associated with the development of ARDS. Additional studies to validate these findings are needed.

## Abbreviations

ALI: acute lung injury
APACHE: Acute Physiology and Chronic Health Evaluation
ARDS: acute respiratory distress syndrome
CSF: cerebrospinal fluid
CT: computed tomography
EPO: erythropoietin
ER: emergency room
GCS: Glasgow Coma Scale
HR: hazard ratio
ICP: intracranial pressure
ICU: Intensive Care Unit
IRB: Institutional Review Board
ISS: Injury Severity Scale
SIRS: systemic inflammatory response syndrome
TBI: traumatic brain injury
VILI: ventilator-induced lung injury
